# Flavonoids as Anticancer Agents

**DOI:** 10.3390/nu12020457

**Published:** 2020-02-12

**Authors:** Dalia M. Kopustinskiene, Valdas Jakstas, Arunas Savickas, Jurga Bernatoniene

**Affiliations:** 1 Institute of Pharmaceutical Technologies, Faculty of Pharmacy, Medical Academy, Lithuanian University of Health Sciences, LT-50161 Kaunas, Lithuania; DaliaMarija.Kopustinskiene@lsmuni.lt (D.M.K.); Valdas.Jakstas@lsmuni.lt (V.J.); 2 Department of Pharmacognosy, Medical Academy, Lithuanian University of Health Sciences, LT-50161 Kaunas, Lithuania; 3 Department of Drug Technology and Social Pharmacy, Faculty of Pharmacy, Medical Academy, Lithuanian University of Health Sciences, Sukileliu pr. 13, LT-50161 Kaunas, Lithuania; Arunas.Savickas@lsmuni.lt

**Keywords:** flavonoids, cancer, ROS, antioxidants, pro-oxidants, mitochondria

## Abstract

Flavonoids are polyphenolic compounds subdivided into 6 groups: isoflavonoids, flavanones, flavanols, flavonols, flavones and anthocyanidins found in a variety of plants. Fruits, vegetables, plant-derived beverages such as green tea, wine and cocoa-based products are the main dietary sources of flavonoids. Flavonoids have been shown to possess a wide variety of anticancer effects: they modulate reactive oxygen species (ROS)-scavenging enzyme activities, participate in arresting the cell cycle, induce apoptosis, autophagy, and suppress cancer cell proliferation and invasiveness. Flavonoids have dual action regarding ROS homeostasis—they act as antioxidants under normal conditions and are potent pro-oxidants in cancer cells triggering the apoptotic pathways and downregulating pro-inflammatory signaling pathways. This article reviews the biochemical properties and bioavailability of flavonoids, their anticancer activity and its mechanisms of action.

## 1. Introduction

Flavonoids are polyphenolic compounds synthesized in plants as bioactive secondary metabolites [1] responsible for their color, flavor and pharmacological activities [2]. The main flavonoid sources are fruits and vegetables [3], and they are also abundant in cocoa products (cocoa powder, chocolate) [4], black and green tea [3,5] and red wine [3,6]. Among the fruits, berries [7,8], plums, cherries [9,10] and apples [10,11] are the richest in flavonoids, whereas tropical fruits are poor in flavonoids [12]. Among the vegetables, the highest levels of flavonoids are found in broad beans [13], olives [14], onions [15], spinach [16] and shallot [17].

Flavonoids are potent antioxidants [11] protecting plants from unfavorable environmental conditions [1], therefore they have attracted attention and have been used in numerous epidemiological and experimental studies to assess their possible beneficial effects in multiple acute and chronic human disorders [18]. In vitro and in vivo studies have shown that flavonoids could exert anti-inflammatory, immunomodulatory [19] and strong anticancer activities [18,20,21].

## 2. Chemical Properties of Flavonoids

**Figure 1:**
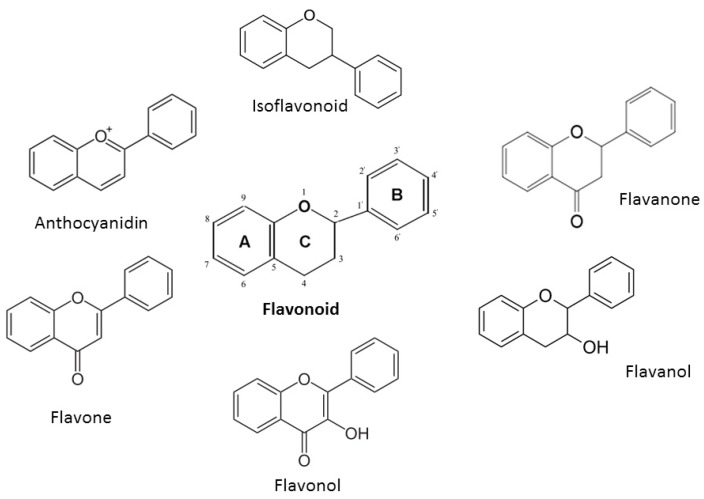
Main chemical structures of flavonoids.

All flavonoids possess the basic flavan skeleton—a 15-carbon phenylpropanoid chain (C6-C3-C6 system), which forms two aromatic rings (A and B) linked by a heterocyclic pyran ring (C) (Figure 1). Based on their chemical structure, degree of oxidation, and linking chain unsaturation flavonoids could be further classified into 6 major groups: isoflavonoids, flavanones, flavanols, flavonols, flavones and anthocyanidins [20,22,23].

**Figure 2:**
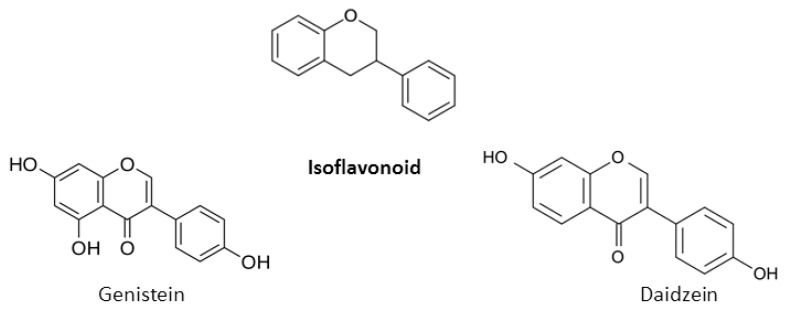
Chemical structures of the main isoflavonoids.

A chromane ring (A and C) is attached to a B ring (Figure 1) at C2 in flavonoids or C3 in isoflavonoids [22]. The main isoflavonoids are genistein and daidzein (Figure 2).

**Figure 3:**
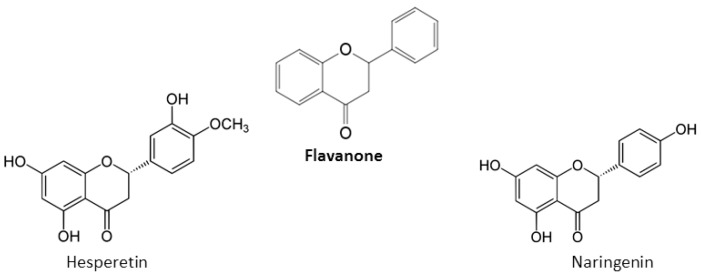
Chemical structures of main flavanones.

A saturated, oxidized C ring is present in flavanones, also described as di-hydroflavones [22]. Main flavanones are hesperetin and naringenin (Figure 3) [22].

**Figure 4:**
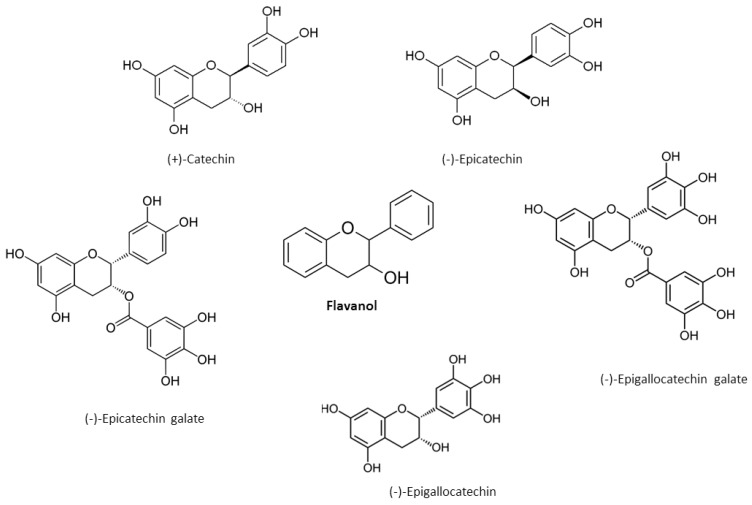
Chemical structures of main flavanols.

A saturated, unoxidized C ring with a hydroxyl group at C3 is common for flavanols, also known as green tea catechins. The most common catechin stereoisomers are cis ((-)-epicatechin) or trans ((+)-catechin according to C2 and C3 position in the molecule [5,24,25]. Flavanols can form gallic acid conjugates epicatechin gallate, epigallocatechin and epigallocatechin gallate during esterification with gallate groups (Figure 4) [5,24,25].

**Figure 5:**
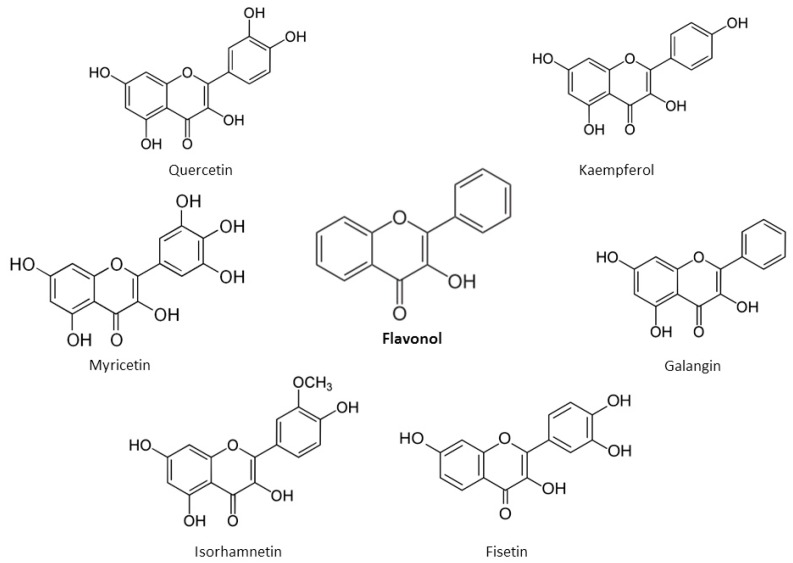
Chemical structures of main flavonols.

Flavonols possess an unsaturated C ring at the C2–C3 position, which is usually hydroxylated at C3 and oxidized at C4 [22]. The main flavonols are quercetin and kaempferol, followed by myricetin, isorhamnetin, fisetin and galangin found in lesser amounts (Figure 5). The –OH moieties in flavonols are responsible for their biological activities.

**Figure 6:**
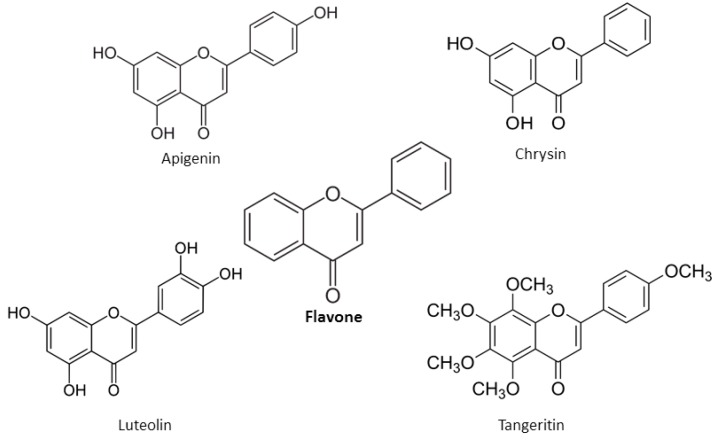
Chemical structures of main flavones.

An unsaturated C ring at C2–C3, non-hydroxylated C3 and a ketonic group at C4 position are present in flavones [22]. The main flavones include apigenin, chrysin, luteolin, and tangeritin (Figure 6).

**Figure 7:**
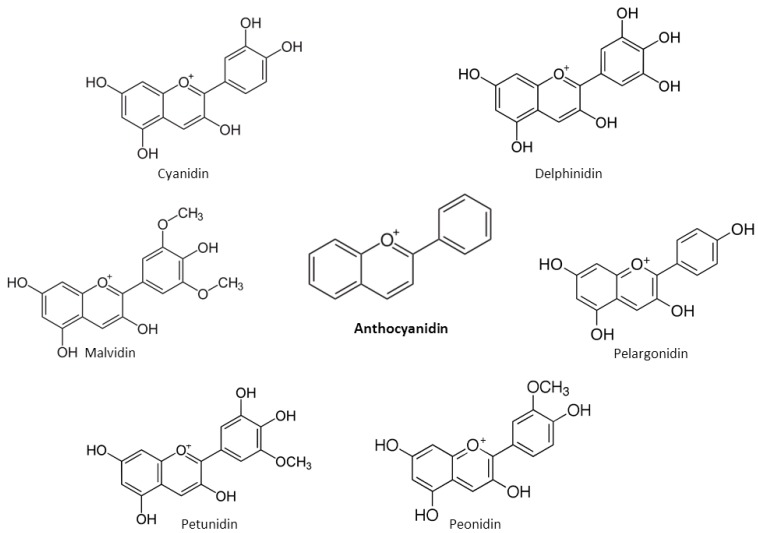
Chemical structures of main anthocyanidins.

Anthocyanidins are water-soluble, unoxidized, unsaturated, flavonoids, mainly found as pH-dependent plant pigments. Anthocyanidins are based on the basic structure of the 2-phenyl-benzopyrylium chromophore–flavylium ion. They are hydroxylated at C3 position and at carbon atoms 3, 4 and 5 in the ring B of the molecule [20]. The main anthocyanidins include cyanidin, delphinidin, pelargonidin, peonidin, petunidin and malvidin (Figure 7) [20].

Flavonoids exist either as glycosides with linked sugars or as aglycones without linked sugars [18,20]. In the cytosol (pH 7.4), flavonoids form a mixture of phenolate anions and neutral phenols. Their proportion depends on the pKa of each phenolic group. Since flavonoids are weak hydrophobic acids, depending on their lipophilicity they have potential to cross cellular and mitochondrial membranes and act as protonophores [26,27,28].

## 3. Bioavailability of Flavonoids

Flavonoids can interact with other nutrients [29,30]: they can decrease glucose absorption due to suppression of carbohydrate-hydrolyzing enzymes (alpha-amylase and alpha-glucosidase) [31] and glucose transporter in the brush border [31]. Fat intake improves flavonoid bioavailability and increases their intestinal absorption via augmented secretion of bile salts which increase micellar incorporation of flavonoids [31]. However, protein intake can decrease flavonoid bioavailability [32,33], affecting both antioxidant efficacy and protein digestibility [32]. The gut microbiome is very important for the absorption and metabolism of flavonoids. After consumption, prior to absorption intestinal or colon microflora are able to hydrolyze glycosylated flavonoids such as flavones, isoflavones, flavonols and anthocyanins into their respective aglycones [33,34]. Aglycones are lipophilic, and therefore passive diffusion is responsible for their pathway to the intestinal epithelial cells while the uptake of glycosides into the intestinal epithelial cells is regulated by the epithelial transporters [34]. After absorption, flavonoids undergo metabolic transformations first in the small intestine, liver and kidney [34]. Methylation, sulfation, or glucuronidation of flavonoids before they reach the circulation and, afterwards, the tissues, could influence their biological activities. Unabsorbed flavonoids remaining in the proximal intestine are further digested in the colon by microbes able to split their heterocyclic oxygen containing ring and the hydroxylated phenyl carboxylic acids formed could be absorbed [34]. The highest concentration of plasma flavonoids in humans usually is reached 1 to 2 h after the intake of flavonoid-rich foods [35]. This depends on the type of flavonoid; for example, catechins and anthocyanins are characterized by a half-life elimination that is 5 to 10 times less compared to flavonols [33]. The concentration of plasma quercetin metabolites are found from 0.7 to 7.6 µM since quercetin is the most abundant dietary flavonoid [35]. Anthocyanins and pro-anthocyanidins have the lowest bioavailability, while quercetin glucosides, catechin, flavanones, isoflavones and gallic acid have the highest bioavailability [18].

## 4. Anticancer Effects of Flavonoids

The ability of flavonoids to scavenge free radicals, regulate cellular metabolism, and prevent oxidative stress–related diseases have been demonstrated in numerous studies [18,19,20,21,36,37]. There is accumulating evidence that many flavonoids exert anticancer activity, however, the molecular mechanisms responsible for this effect have not been fully elucidated yet.

**Figure 8:**
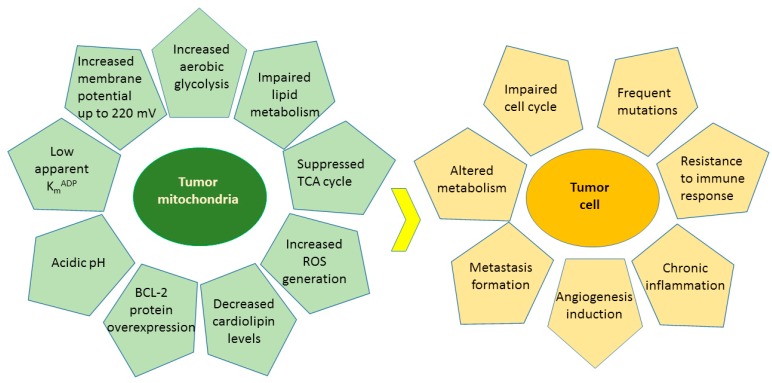
The main characteristics of tumor mitochondria and tumor cells.

Cancer is a heterogeneous disease characterized by uncontrolled proliferation and impaired cell cycle leading to the growth of abnormal cells that invade and metastasize to other parts of the body [38,39]. Oxidative stress, hypoxia, genetic mutations and lack of apoptotic function are the main internal causes of cancer, whereas the external causes are related to increased exposure to stress, pollution, smoking, radiation and ultraviolet rays [40]. Altered metabolism, impaired cell cycles, frequent mutations, resistance to immune response, chronic inflammation, formation of metastasis, and induction of angiogenesis are the main characteristics of the cancer cells [38] (Figure 8). There is emerging evidence that cancer is a metabolic disease determined by various degrees of mitochondrial dysfunctions and metabolic alterations [38,39,41]. Mitochondria play essential roles in cellular energy supply, regulation of metabolism, cell death signaling and reactive oxygen species (ROS) generation. The main metabolic alterations of the tumor cells involve increased aerobic glycolysis [42], deregulated pH [43], impaired lipid metabolism [44], increased generation of ROS [45], and compromised enzyme activities [38,46] (Figure 8). As a direct consequence, the extracellular environment becomes acidic and more favorable to inflammation [47], glutamine-driven lipid biosynthesis increases and upregulates the pathways involved in tumorigenesis initiation and metastasis [48], cardiolipin levels decrease in membranes causing impaired enzyme activities [49,50,51], mitochondria are hyperpolarised [38], and this effect correlates with the malignancy and invasiveness of cancer cells [38].

Flavonoids exert a wide variety of anticancer effects: they modulate ROS-scavenging enzyme activities, participate in arresting the cell cycle, induce apoptosis, autophagy, and suppress cancer cell proliferation and invasiveness [18,19,20,21,36,37].

### 4.1. Flavonoids in Oxidative Stress

**Figure 9:**
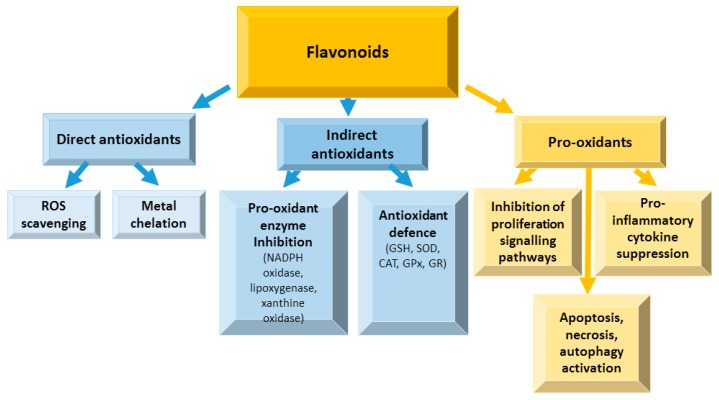
Antioxidant and pro-oxidant activities of flavonoids in oxidative stress. ROS—reactive oxygen species, NADPH-oxidase—nicotinamide adenine dinucleotide phosphate oxidase, GSH—glutathione, SOD—superoxide dismutase, CAT—catalase, GPx—glutathione peroxidase, GR—glutathione reductase.

When the cellular homeostasis between the pro-oxidant activities and antioxidant defense is impaired, the production of ROS increases, and free radicals accumulate [18]. ROS are mainly generated in the electron transport chain in mitochondria as the byproducts of oxidative phosphorylation in the cell [52]. The amount of ROS produced causes oxidative stress which is involved in the development of inflammation processes leading to many degenerative diseases and cancer. Flavonoids have dual action regarding ROS homeostasis—they act as antioxidants under normal conditions and are potent pro-oxidants in cancer cells triggering the apoptotic pathways [53,54] (Figure 9). 

Flavonoids can directly scavenge ROS, and chelate metal ions [55] due to their ability stabilize the free radicals due to the presence of phenolic hydroxyl groups [56]. Indirect flavonoid antioxidant effects are related to activation of antioxidant enzymes, suppression of pro-oxidant enzymes, and stimulating production of antioxidant enzymes and phase II detoxification enzymes [55]. Both antioxidant and pro-oxidant activities are involved in flavonoid anticancer effects [57,58].

Isoflavone genistein promoted breast cancer cell arrest at G2/M phase and subsequent ROS dependent apoptosis [59]. Daidzein promoted apoptosis in breast cancer MCF-7 cells due to the ROS generation [60]. Flavanone hesperetin induced apoptosis of gall bladder carcinoma [61], esophageal cancer [62], hepatocellular carcinoma [63] and human breast carcinoma MCF-7 cells [64] via activating the mitochondrial apoptotic pathway by increasing the ROS production. Flavanone naringenin exerted anti-cancer effects on choriocarcinoma JAR and JEG 3 cell lines by inducing the generation of ROS and activation of signaling pathways [65]. It also initiated an apoptotic cascade in human epidermoid carcinoma A431 cells [66]. In prostate cancer PC3 and LNCaP cell lines, naringenin suppressed proliferation and migration and induced apoptosis and ROS generation [67]. Furthermore, naringenin reduced ROS generation and enhanced the activity of superoxide dismutase, catalase, glutathione in chronic diseases and cancer [68]. Cocoa catechins and procyanidins have been shown to induce apoptotic morphological changes, DNA damage and apoptosis in epithelial ovarian cancer cells due to their prooxidant properties [69]. Cocoa polyphenolic extract activated the ERK1/2 pathway, thus increasing the activities of glutathione peroxidase and reductase in HepG2 cells [70]. Cocoa catechins and procyanidins also protected Caco2 cells against an induced oxidative stress and subsequent cellular death by reducing ROS production [71]. Due to antioxidant properties, cocoa flavanols exerted beneficial effects in the protection from colon cancer [72,73]. Flavonol quercetin exerted potent cancer chemopreventive properties [74,75]. Recent studies showed that quercetin reduced the proliferation of hepatocellular carcinoma HepG2 cells decreasing the intracellular ROS level [76]. It increased ROS production and the apoptotic cell number in human gastric cancer AGS [77] and human breast cancer MCF-7 cells [78]. Flavonol kaempferol inhibited the growth of cancerous bladder cells due to ROS level modulation-induced apoptosis and S phase arrest [79]. It activated caspases due to ROS generation and stimulated apoptosis in colorectal cancer HCT116, HCT15, and SW480 cell lines [80]. Furthermore, kaempferol exerted cytotoxic effects on rat hepatocellular carcinoma cells via ROS-mediated mitochondrial targeting [81]. The anticancer activities of flavones apigenin and luteolin in ovarian cancer cell lines (A2780, OVCAR-3 and SKOV-3) were also related to the changes in ROS signaling, as well as to the promotion of apoptosis [82,83]. Moreover, apigenin activated apoptosis also in human cervical cancer-derived cell lines including HeLa (human papillomavirus/HPV 18-positive), SiHa (HPV 16-positive), CaSki (HPV 16 and HPV 18-positive), and C33A (HPV-negative) cells due to increased ROS generation and launched mitochondrial apoptotic pathways [84]. Flavone chrysin was reported to augment ROS and lipid peroxidation levels, leading to the death of choriocarcinoma (JAR and JEG3) [85], bladder cancer [86] and ovarian cancer (ES2 and OV90) cells [87]. The antioxidant activity of flavonoids was also investigated in humans. It was found that serum total antioxidant capacity correlates with anthocyanin consumption in the diet [88]. Furthermore, cyanidin induced cell death via ROS modulation in the DU145 and LnCap human prostatic cancer cells [89]. Cyanidin and delphinidin accelerated cellular ROS accumulation, suppressed glutathione reductase, and depleted glutathione resulting in cytotoxicity in metastatic (LoVo and LoVo/ADR) colorectal cancer cells [90].

Thus, numerous studies show beneficial effects of flavonoids as potent antioxidants under normal and pro-oxidants under pathological conditions, capable of activating apoptosis and suppressing proliferation and inflammation.

### 4.2. Flavonoids in Apoptosis

**Figure 10:**
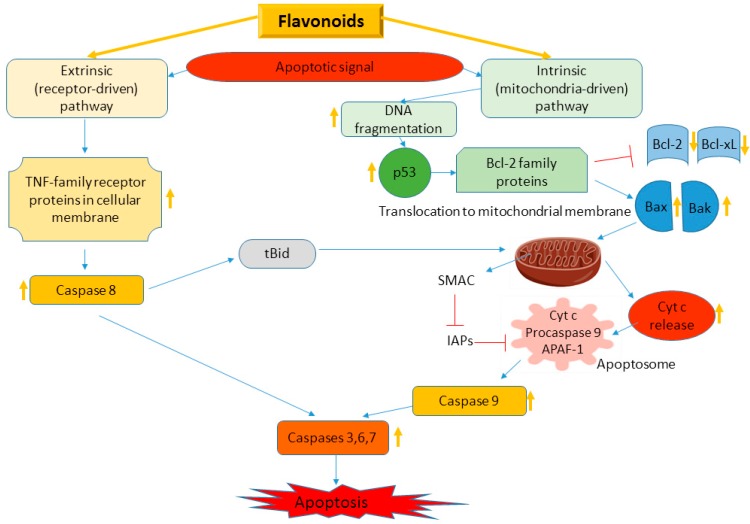
Flavonoid targets in extrinsic and intrinsic apoptosis pathways. TNF—tumor necrosis factor, tBid—truncated Bid, Bcl-2—B-cell lymphoma protein 2, Bcl-xL—Bcl-2 homologue splice variants, Cyt c—cytochrome c, SMAC—second mitochondrial activator of caspases, IAPs—inhibitor of apoptosis proteins, APAF-1—apoptotic protease activating factor 1. Yellow arrows show the effect of flavonoids (activation or suppression).

Cancer cells are resistant to apoptosis—a programmed cell death, usually induced by a series of signal transduction pathways and pro-apoptotic proteins—caspases and Bcl-2 family proteins [20,91]. There are two main signaling cascades of apoptosis—extrinsic, related to tumor necrosis factor (TNF) superfamily with main signaling protein—caspase 8; and intrinsic—mitochondrial pathway, where Bcl-2 family proteins launch the activation of caspases 9, 3 and 7 (Figure 10) [20,91]. There is an overexpression of oncogenic genes (e.g., *c-Myc*), leading to cellular proliferation and p53 suppression, and activated anti-apoptotic proteins of Bcl-2 family in cancer cells [92], whereas pro-apoptotic proteins and caspases could be downregulated [91,92]. Flavonoids could target apoptotic signaling cascade stimulating the cell death pathways [20,21] (Figure 10).

Flavonoids acting as pro-oxidants could suppress proliferation of cancer cells by inhibition of epidermal growth factor receptor/mitogen activated protein kinase (EGFR/MAPK), phosphatidylinositide 3-kinases (PI3K), protein kinase B (Akt) as well as nuclear factor kappa-light-chain-enhancer of activated B cells (NF-κB) [18,20,38].

Isoflavonoid genistein could regulate estrogen receptor-α expression and change Bax/Bcl-2 ratio downregulating proliferation, differentiation, and activating apoptosis in MCF-7 and 3T3-L1 cells [93]. Moreover, genistein suppressed Bcl-2, Bcl-xL, c-inhibitor of apoptosis protein 1 (c-IAP1), survivin, and NF-κB in C200 and A2780 cells [94], increased caspase-3 activity in HT-29 colon cancer cells [95] and activated intrinsic apoptotic signaling pathway in HCT-116 and LoVo cells [96]. Isoflavonoid daidzein also acted as phytoestrogen [97]. It promoted cytochrome c release from mitochondria, leading to caspase 7 and 9 activation and also altered Bax/Bcl-2 ratio in MCF-7 cells [60,98]. Daidzein induced apoptosis in the HCCSK-HEP-1 cell line via Bak upregulation and downregulation of anti-apoptotic proteins, resulting in cytochrome c release from mitochondria and activating subsequent apoptotic pathway involving caspases 3 and 9 [99]. Flavanone hesperetin induced cytochrome c release, activation of caspases-3 and -9, and reduced Bax to Bcl-2 ratio in gastric cancer cells [100], in the Eca109 cell line [62] as well as in the HT-29, MCF-7, and MDA-MB-231 cell lines [64,101]. In H522 cells, hesperetin induced extrinsic apoptotic pathway due to overexpression of TNF-protein superfamily members, caspase-9 activation, and decrease in p53 level [102]. Furthermore, hesperetin inhibited the NF-κB signaling pathway and reduced Bcl-2 transcription and translation in PC-3 cells [103]. Flavanone naringenin could induce apoptosis via increased p53 expression, Bax and caspase-3 cleaving, and downregulated Bcl-2 and survivin in SGC-7901 cell line [104,105]. Naringenin-induced extrinsic apoptotic pathway was related to overexpression of TNF-family proteins [20]. Flavanols catechins, especially epigallocatechin galate, induced apoptosis and cell-cycle arrest, inhibited NF-κB, leading to cyclooxygenase-2 (COX) overexpression [106]. Moreover, it increased Bax/Bcl-2 ratio, upregulated p53, p21, caspases-3, and -9, and down-regulated PI3K, Akt, and Bcl-2 in T47D and HFF cells [107]. Catechins could also alter the expression of anti- and pro-apoptotic genes [108,109,110]. Cocoa flavanols have been shown to moderate apoptosis pathways in HepG2 [111,112] and Caco-2 cells [72]. Flavonol quercetin, a widely abundant phytoestrogen [20], was able to induce intrinsic apoptotic pathway via Bax and caspase-3 upregulation and downregulation of Bcl-2 in MCF-7 cells [113,114,115]. Quercetin activated apoptosis in PC-3 and LNCaP cells regulating the p53 signaling pathway [116]. In HL-60 cells, quercetin activated intrinsic apoptotic cascade-modulating COX-2, activating caspase-3, modulating Bax, Bad, Bcl-2 expression and inducing cytochrome c release from mitochondria [117]. In a human hepatoma cell line, quercetin induced apoptosis via caspase activation, regulation of Bcl-2, and inhibition of PI-3-kinase/Akt and extracellular-signal-regulated kinase (ERK) pathways [118]. Quercetin was also able to suppress cancer cell proliferation due to inhibition of PI3K/Akt pathway [119]. Flavonol kaempferol, a phytoestrogen [120], induced intrinsic apoptosis in A2780/CP70, A2780wt and OVCAR-3 cell lines. Its main effects were related to the activation of caspases 3 and 7, the upregulation of p53, Bax and Bad and the downregulation of Bcl-xL protein [121]. In HeLa cells, kaempferol activated apoptosis elevating the Bax/Bcl-2 ratio [122]. Flavone apigenin also was reported to have estrogenic activity [123]. In PC-3 and DU145 cell lines apigenin induced Bax overexpression, the downregulation of Bcl-2 and Bcl-xL proteins, and stimulated cytochrome c release from mitochondria and subsequent activation of signaling cascades [124,125]. Apigenin upregulated p53 in ACHN, Caki-1 RCC cell lines [126]. In T24 cell line, apigenin inactivated PI3K/Akt signaling pathway, activated the intrinsic apoptotic pathway, promoted the cytochrome c release from mitochondria, inhibited Bcl-xL [127,128]. In HCT-116 cells, apigenin activated both extrinsic and intrinsic apoptotic pathways [129]. Flavone chrysin activated apoptosis in HeLa cells due to increased DNA fragmentation and stimulated p38 and NF-κB pathways [20]. Chrysin upregulated caspase 3 in the U937 cell line [130]. In SP6.5 and M17 melanoma cells, chrysin induced the intrinsic apoptotic pathway due to cytochrome c release-driven activation of caspases 3 and 9 [131]. Anthocyanidin pelargonidin stimulated the cytochrome c release from mitochondria, activated Bax, Bid, caspases 3 and 9, and inhibited the expression of Bcl-2 and Bcl-xL in HT-29 cells [132,133]. Furthermore, pelargonidin downregulated the PI3K/Akt signaling pathway thus suppressing proliferation of U2OS cell line [132]. Cyanidin could activate cytochrome c and upregulate Bax protein expression [20]. In U87 cells, cyanidin promoted Bax and p53 expression and downregulated Bcl-2 expression [134]. Delphinidin decreased proliferation of the SKOV3 cell line due to the suppression of PI3K/Akt and ERK1/2/MAPK signaling pathways [135]. Delphinidin was also able to activate caspases 3 and 9 in the NSCLC cell line [136].

Thus, flavonoids could activate cell death signaling pathways in cancer cells by a dual mechanism—activating anti-apoptotic proteins and suppressing pro-apoptotic proteins and caspases.

### 4.3. Immunomodulatory and Anti-Inflammatory Effects of Flavonoids

**Figure 11:**
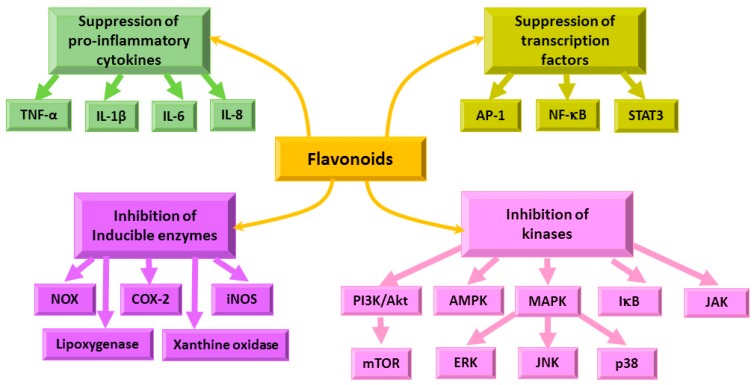
Flavonoid targets during inflammation processes. TNF—tumor necrosis factor, IL—interleukin, AP-1—activator protein 1, NF-κB—nuclear factor kappa-light- chain-enhancer of activated B cells, STAT3—signal transducer and activator 3, NOX—NADPH oxidase, COX-2—cyclooxygenase-2, iNOS—inducible nitric oxide synthase, AMPK—AMP—activated protein kinase, PI3K—phosphatidylinositide 3-kinases, Akt—protein kinase B, mTOR—mammalian target of rapamycin, MAPK—mitogen activated protein kinase, ERK—extracellular-signal-regulated kinase, JNK—c-Jun N-terminal kinase, p38—p38 kinse, IκB—IκB kinase, JAK—Janus kinase.

Chronic inflammation leads to tumor development, modulating cellular transformation, survival, proliferation, invasion, metastasis, and angiogenesis pathways [137]. Flavonoids were shown to exert anti-inflammatory action via immune cell regulation, suppression of chemokines, COX-2, cytokines and pro-inflammatory transcription factors, inhibition of PI3K/Akt, inhibitor of kappa kinase/c-Jun amino-terminal kinases (IKK/JNK) [19,21,137]. The NF-kB signaling pathway is crucial in the regulation of inflammation [19,21,137] and is related to the modulation of a wide variety of oncogenes (Figure 11) [137].

The immune system is a key player in protecting an organism from infections and cancer. B and T lymphocytes and macrophages are the major cells responsible for the immunity. B cells secret antibodies which are able to attach to pathogens, marking them so they are recognized and destroyed by phagocytes [138,139]. T cytotoxic cells are able to kill tumor cells directly, and T helper cells secrete cytokines and mediators which regulate the activities of B lymphocytes and macrophages [138,139]. Flavonoids have been shown to modulate directly the differentiation and count of the cells belonging to the immune system [138,139]. Furthermore, flavonoids can inhibit the activity of the mammalian target of rapamycin (mTOR) and thus reduce T effector differentiation and induce T regulatory cells [140]. Programmed cell death protein 1 (PD-1) is present on the surface of B cells, T cells and macrophages [141]. When programmed death-ligand 1 (PD-L1)—a protein present on the surface of tumor cells—binds the PD-1, the signal is sent to suppress the immune system response; therefore, the inhibitors of PD-L1/PD-1 signaling pathway could be promising agents in cancer immunotherapy [141]. The studies on flavonoids as possible suppressors of PD-L1/PD-1 immune checkpoint have not been very intense yet, nevertheless the first encouraging results were obtained demonstrating the inhibition of PD-L1 expression by flavone apigenin in A375 melanoma cells [142] and PD-1/PD-L1 inhibition in vitro by flavonols quercetin [143] and fisetin [143] as well as isoflavonoid glyasperin C [144].

Isoflavone genistein has been shown to modulate the expression of several genes involved in cell cycle regulation, migration, inflammation, and the PI3K and MAPK pathways in HeLa cells [145]. Genistein exerted influence on the expression of inflammatory-related genes in breast cancer MCF-7 (high ERα/ERβ ratio), T47D (low ERα/ERβ ratio), and MDA-MB-231 (ERα-negative) cell lines [146]. Furthermore, genistein inhibited the increased M2 polarization of macrophages and stemness of ovarian cancer SKOV3 and OVCA-3R cell lines by the co-culture of macrophages with ovarian cancer stem-like cells through disrupting the interleukin (IL)-8/STAT3 signaling axis [147]. Isoflavone daidzein downregulated the pro-inflammatory NF-kB and JNK signaling pathways in adipocyte and macrophage co-cultures [148]. Flavanone hesperetin suppressed secretion of TNF-α, IL-6, and IL-1β; decreased inducible nitric oxide synthase (iNOS) and COX-2 gene expression; down-regulated NF-κB (p65) phosphorylation in lipopolysaccharide -induced RAW 264.7 cells [149]. Hesperetin inhibited cell proliferation markers, angiogenic growth factors, COX-2 mRNA expression in 1,2-dimethylhydrazine-induced colon cancer [150].

Quercetin and naringenin prevented the lowered mRNA expression of liver IL-4, p53 and Bcl-2 in a diethylnitrosamine/2-acetylaminofluorene-induced hepatocarcinogenesis model in rats [151]. Naringenin inhibited the migration of breast cancer MDA-MR-231 cell line via modulation of inflammatory and apoptotic signaling pathways [152]. It also suppressed the migration and invasion of glioblastoma cells due to inhibition of ERK and p38 activities [153]. Catechins, especially epigallocatechin galate, inhibited NF-κB pathway and suppressed COX-2 overexpression [106]. Epicatechin induced NF-κB, AP-1 and Nrf2 via PI3K/AKT and ERK signalling in HepG2 cells [112]. Cocoa polyphenols prevented inflammation in the colon of azoxymethane-treated rats and in TNF-α-stimulated Caco-2 cells [154]. Flavonol quercetin inhibited the expression of matrix metallopeptidases MMP9 and MMP2 in human glioblastoma U251 cell line [155]. In ascite cells of Dalton’s lymphoma-bearing mice, quercetin downregulated the phosphorylation of Akt and PDK1 resulting in suppressed phosphorylation of downstream survival factors such as Bad, glycogen synthase kinase-3 (GSK-3β), mTOR, and nuclear factor of kappa light polypeptide gene enhancer in B-cells inhibitor alpha (IkBα) [156]. Furthermore, quercetin attenuated the levels of angiogenic factor vascular endothelial growth factor A (VEGF-A) and inflammatory enzymes COX-2 and iNOS [156]. Quercetin inhibited the migration and invasion of the human colon cancer Caco-2 cell line via regulation of the toll-like receptor 4 (TLR4)/NF-kB pathway [157]. Quercetin has been shown to be a potent inhibitor of mTOR activity and the PI3K/Akt signaling pathway in cancer cells [158]. Flavonol kaempferol downregulated TNF-alpha induced IL-8 promoter activation and gene expression in HEK 293 cells [159]. Furthermore, kaempferol reduced the plasma levels of the cytokines IL-6, IL-1β and TNF-α and suppressed the MAPK and NF-κB signaling pathways [160,161,162]. Flavone apigenin downregulated TNF-α-related inflammatory signaling in the A375 human melanoma cell line [163]. Apigenin decreased myeloperoxidase (MPO), inflammatory cytokine and COX-2 levels and downregulated NF-κB and STAT3, thereby inhibiting inflammation and inflammation-induced carcinogenesis in an inflammatory bowel disease and colitis-associated cancer model [164]. Apigenin could suppress Akt, ERK, MAPK, COX-2, IL-6, TNF-α, IL-1, iNOS activities in vitro and in vivo [163,165]. Flavone chrysin inhibited iNOS and COX-2 expression, and decreased the levels of proinflammatory cytokines IL-6, TNF-α, and prostaglandin E(2) (PGE(2)) in a renal cancer model in rats [166]. Cyanidin has been shown to inhibit pro-inflammatory cytokine interleukin-17A (IL-17A) [167]. Pelargonidin suppressed the production of TNF-α or IL-6 and the activation of NF-κB or ERK½ in vitro [168]. Cocoplum anthocyanins inhibited the production of TNF-α, IL-6 and the activation of NF-κB or ERK ½ in HT-29 colorectal adenocarcinoma cells [169]. Delphinidin suppressed the activation of NF-κB through MAPK signaling pathways in MCF-7 human breast carcinoma cells [170].

Chronic inflammation often precedes tumor development, therefore anti-inflammatory effects of flavonoids could be very important in decreasing the inflammation and enhancing the antitumor activity of immune cells.

### 4.4. Effects of Flavonoids on Mitochondrial Functions

Tumor-cell metabolism is altered compared to normal cells due to highly abnormal mitochondrial functions (Figure 8) [171]. Therefore, recent interest in natural compounds reverting the mitochondria to normal mode has emerged, and flavonoids have also been tested among potential drug candidates [20,36,37].

**Figure 12:**
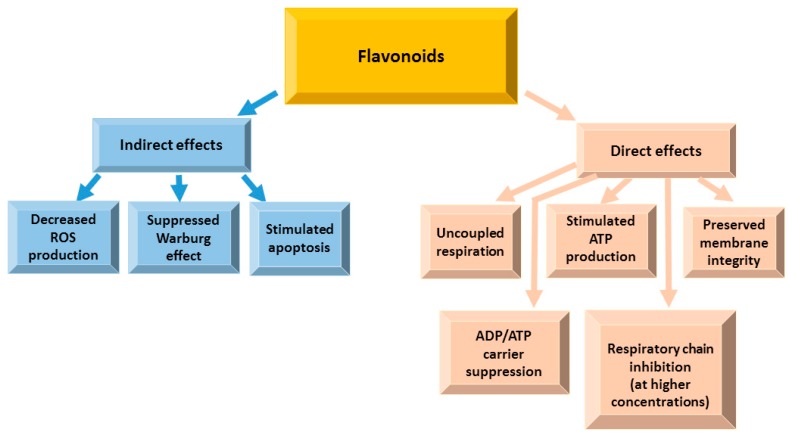
Indirect and direct effects of flavonoids on mitochondrial functions.

Hexokinase and voltage-dependent anion channel (VDAC) coupling in mitochondria prevents induction of apoptosis in tumors [14]. In human breast carcinoma (MDA-MB-231 and MCF-7) cells, an O-methylated flavone oroxylin A was reported to promote the detachment of hexokinase from mitochondria, resulting in inhibition of glycolysis [17]. Overexpression of antiapoptotic proteins of the BCL-2 family in mitochondria results in resistance to apoptotic pathways [37]. Flavanone hesperetin reduced antiapoptotic BCL-2 family protein transcription and translation in the human prostate cancer PC-3 cell line [103]. Naringenin and epigallocatechin-3-gallate decreased the BCL-2 expression accordingly in gastric cancer (SGC-7901) cells [104] and in cholangiocarcinoma (HuCC-T1) cells [172]. Mitochondrial adenine nucleotide translocase is a protein embedded in the mitochondrial inner membrane and responsible for ATP/ADP exchange [173]. It is one of the component of mitochondrial permeability transition pore complex, which is a key factor triggering apoptosis [174]. Quercetin (50 µM) was able to inhibit adenine nucleotide translocase by 46% in mitochondria isolated from rat kidney cortex [175], whereas apigenin (20 µM) inhibited it in human prostate cancer DU145 cells [176]. A procyanidin-rich French maritime pine (*Pinus pinaster*) bark extract inhibited the electron transport chain in isolated rat liver mitochondria and in submitochondrial particles, affecting complexes I, II and III [177]. An isoflavone genistein induced mitochondrial permeability transition in isolated rat liver mitochondria due to increased ROS generation at the complex III of the mitochondrial respiratory chain [178]. Epigallocatechin-3-gallate suppressed the growth of highly aggressive malignant pleural mesothelioma cells inhibiting complex I, II, and ATP synthase [179]. Moreover, epigallocatechin-3-gallate modulated mitochondrial bioenergetic functions and regulated apoptosis signaling cascade [180]. Anthocyanins were able to reduce cytosolic cytochrome c preventing apoptosis and support the electron transfer between NADH dehydrogenase and cytochrome c [181,182]. The inhibition of the tricarboxylic acid (TCA) cycle is one of the hallmarks of cancer [37]. Quercetin [183], kaempferol [184], hesperetin and naringenin [185] have been shown to stimulate the TCA cycle shifting anaerobic glycolysis to oxidative phosphorylation, normally suppressed in cancer cells. The effects of flavonoids on mitochondrial functions are summarized in Figure 12.

In tumor cells, mitochondria are usually hyperpolarized, and their membrane potential reaches 220 mV [186,187] making them resistant to cell death signaling. Our group evaluated direct effects of selected flavonoids on the functions of cardiac mitochondria respiring on pyruvate and malate as substrates [188,189,190,191]. The results demonstrated that (-)-epicatechin [190,191], procyanidin B2 [190,191], hyperoside [189,190], quercetin [189,190], quercitrin [189,190] and rutin [189,190] uncoupled oxidation from phosphorylation. Furthermore, all flavonoids were reported to induce apoptosis (reviewed in [20]) and initially decrease mitochondrial membrane potential [20].

Most flavonoids have pKa values ranging between 6 and 9, i.e., close to the physiological pH of the cytosol and mitochondrial compartments, and favorable distribution coefficients [26,27,28], and therefore they have the ability to reach the mitochondrial matrix and release a proton in its relatively basic environment (pH 7.8). This effect might be crucial in the chemoprevention of cancer since the mild mitochondrial uncoupling effectively protects cells from oxidative stress.

### 4.5. Effects of Flavonoids on Gut Microbiota

The gastrointestinal tract, and especially the intestinal barrier, is very important in sustaining health [192,193]. Intestinal epithelium, besides nutrient absorption, provides a barrier controlling the entrance of microorganisms, their metabolic products and toxins as well as toxins present in ingested foods [194]. Due to anti-inflammatory action, flavonoids could protect the integrity of the intestinal barrier [192,195,196]. Flavanol epicatechin and flavonol quercetin suppressed systemic inflammation in rodent models of overfeeding (high fructose and high fat diets) [197,198,199]. Plant extracts rich in anthocyanins and pure anthocyanins could protect Caco-2 cell monolayers from permeabilization due to inflammation [200,201], whereas O-glucosides of delphinidin and cyanidin were more potent than the O-glucosides of petunidin, peonidin and malvidin [200]. In several metaanalysis, flavones, flavanols, flavonols, isoflavones, anthocyanidins and proanthocyanidins could reduce colorectal cancer risk [202,203,204,205]. Thus, some flavonoids were able to prevent and cure metabolic diseases directly at the gastrointestinal tract [206]. 

Flavonoids could suppress the activity of gut metabolizing enzymes—α-glucosidase, pancreatic lipoprotein lipase and amylase [192]. In vivo, proanthocyanidins inhibited triglyceride absorption in mice and in humans [207]. Oolong tea-derived epigallocatechin galate suppressed α-amylase [208]. In the gastrointestinal tract lumen, the decreased activities of α-glucosidase, pancreatic lipoprotein lipase and amylase would lead to a suppressed absorption of glucose from complex carbohydrates and fatty acids from triglycerides [192,209].

The microbiota present in the intestinal lumen is very important for the whole body. The link between pathological conditions, ingested food and the gut microbiota has not been established yet, although the primary investigations let hypothesize that it might be possible to prevent chronic diseases by modulating the intestinal microflora [210,211,212]. Most flavonoids (except flavanols) are naturally attached to sugars as β-glycosides, therefore they are not readily absorbed in the small intestine [213,214], and glycosylated flavonoids reach the colon [34] where the microbiota digest the flavonoids forming phenolic acids and other metabolites, which can later be absorbed [213,215]. Thus, flavonoids in the colon could influence the gut microbiome, whereas microbes could modulate flavonoid activity and bioavailability metabolizing them and these processes may be beneficial for health [192]. Flavonoids are known to exert antimicrobial activity, inhibiting specific microbes, such as pathogenic and commensal microorganisms [209]. Quercetin was shown to suppress the growth of *Lactobacillus* sp., *Bacteroides galacturonicus* and *Ruminococcus gauvreauii* [216]. The polyphenols present in cloudberry could decrease the growth of *Candida albicans*, *Bacillus cereus*, *Helicobacter pylori*, *Campylobacter jejuni*, *Staphylococcus epidermidis*, *Staphylococcus aureus* and *Clostridium perfingen*s [217]. Furthermore, flavonoids could promote the growth of specific microbes in the gut [192]. Mice ingesting food rich in apple flavonoids demonstrated higher levels of bacteria belonging to a combined group of *Bacteroides–Prevotella–Poryphyromonas* and *Bifidobacterium* spp. but significantly decreased levels of *Lactobacillus* spp. [218]. Quercetin and rutin increased the growth of *Bifidobacterium bifidum* in vitro [219]. These studies show that flavonoids can affect microbial populations by changing endotoxin production, converting primary into secondary bile acids [220], sustaining immune homeostasis [221] and participating in bioactive and nutrient absorption and metabolism, thereby regulating short-chain fatty acid formation [222].

Thus, ingestion of flavonoids is related to the suppression of inflammatory markers via the downregulation of the transcription factor NF-κB signaling pathway in the gastrointestinal tract that could be a promising strategy in therapeutic approaches preventing chronic diseases and controlling inflammation due to the modulation of the microbiota. However, at high doses flavonoids could exert pro-oxidant properties, act as mutagens and inhibit enzymes involved in hormone metabolism [223,224,225]. Since adverse effects due to flavonoid overdose may outweigh the beneficial activities, the excessive intake of flavonoids in diets should be avoided [224,225]. 

## 5. Conclusions and Future Perspectives

Flavonoids are natural molecules, present in human foods and beverages since ancient times; therefore, they do not have dangerous side effects as synthetic anti-cancer drugs Numerous studies have shown their strong positive activities in reducing inflammation, modulating immune response, and supporting and restoring the normal functions of cells. Flavonoids exert a wide range of anticancer effects and, therefore, they could serve as potential compounds for further studies on the development of novel cancer chemopreventive agents and on understanding their detailed mechanisms of action. Furthermore, the daily intake of flavonoids as flavonoid-rich foods or flavonoid supplements could induce favorable changes in the gut microbiota, decreasing the risk of cancer and normalizing vital functions at cellular level.
